# Supplementary material for: Glycerol enhances mitochondrial metabolism and inflammatory response in pro-inflammatory macrophages
Source: EMBO Rep. 2026 Apr 14;27(10):2614–38. doi: 10.1038/s44319-026-00747-y (PMC13219733; doi:10.1038/s44319-026-00747-y)
Supplement: Supplementary file 1 — Appendix [file 44319_2026_747_MOESM1_ESM.pdf]

Extracellular glycerol enhances mitochondrial metabolism and inflammatory responses in pro-inflammatory macrophages

Appendix

Content：

Appendix Table S1.
Summary of experiments on the effects of glycerol on macrophages --------- 2
Appendix Table S2.
List of upregulated genes identified by RNA-seq ---------------------------------- 3
Appendix Table S3.
List of downregulated genes identified by RNA-seq ------------------------------ 4
Appendix Table S4.
Gene list of mitochondrial metabolic pathways for heatmap visualization --- 5
Appendix Figure S1.
Uncropped immunoblotting image and FACS gating ----------------------------- 6

Appendix Table S1. Summary of experiments on the effects of glycerol on macrophages

Experiment	Figure	Mf	Glycerol treatment time	Glycerol concentration
**Effect of Glycerol on LPS-primed macrophages**				
^{14}C Glycerol uptake	Fig. 1A, 1B	M1	3 min	1μM-10mM
^{13}C metabolomic analysis	Fig. 1C, 1D	M1	3 hr	1mM, 10mM
Flux analysis	Fig. 2A-2G	M1	3, 24hr	1mM
RNA seq analysis	Fig. 3B	M1	6 hr	1mM
RNA expression (ETC)	Fig. 3C-3F	M1	3, 6, 24hr	1mM
ChIP assay (inflam.)	Fig. 4A	M1	3 hr	1mM
RNA expression (inflam.)	Fig. 4B	M1	24 hr	1mM
**Effect of low-dose glycerol on LPS-primed macrophages**				
Flux analysis	EV2H	M1	3 hr	100-300μM
RNA expression (inflam.)	EV3C	M1	24 hr	100μM-1mM
**Effect of glycerol on M0 macrophages**				
Flux analysis	EV2I	M0	3 hr	100μM-1mM
RNA expression (inflam.)	EV3D	M0	24 hr	1mM
RNA expression (ETC)	EV3B	M0	6hr	1mM

"M1" denotes macrophages primed with LPS for 24 h (used here as an operational term for LPS-primed macrophages).
inflam.: inflammation-related genes
ETC: ETC complex I–III genes

Appendix Table S2. List of upregulated genes identified by RNA-seq

Upregulated gene							
Ctsd	H3f3b	Psmb4	Rpl36a	Tspo	Snrnp70	Clic1	Pggt1b
Cyba	Rps21	Snrpd2	Mxd3	Erp29	Gm2000	Polr2g	Lmna
Atp6v0b	Cox6a1	Rpl18	Snf8	Hmox1	Eif5a	Atp5md	Slc15a4
Gpx4	Cox4i1	Cox5a	Psmb3	B3gat3	Pnkp	Pam16	Mrps12
Ckb	Uqcr10	Aprt	Edf1	Rabac1	Ccdc85b	Batf3	Sdc4
Atp5e	Prdx2	Atp5d	Cox6b1	Rpl34	Atp5k	Zc3hc1	Rps27
Rps15a	Cd52	Ndufb10	Saa3	C1qa	Sash3	Cox7a2	Ndufv2
Gpx1	Rad9a	Fam166a	Sem1	Cd37	Ocel1	Mrpl24	Phox2a
Rpsa	Tuba1b	Rpl9	Tyrobp	Cox8a	Acat3	Micos13	Psma6
Rpl19	Msrb1	Dnase2a	Prdx1	Psme2	Anxa2	Jund	H2-Ab1
Eif5b	Vamp8	Cd63	Fis1	H2-Q7	Coa3	Trmt112	Smim20
Rps14	Tuba1c	Cst3	Rps29	Lamtor4	Capn12	Psen2	Mpnd
Prdx5	Metrnl	Cxcl10	Mrpl12	Ifi30	Rnaseh2c	Sf3b5	Imp3
Uqcrq	Coro1a	Ppp1r14b	mt-Nd6	Plekhj1	Lat2	Emc8	Msto1
Myl6	Rpl36	Clpp	Loxl3	Pebp1	E2f1	Btg1	Gngt2
Ndufa2	Tnf	Rpl26	Psmb8	Uqcr11	Cmtm7	Ccdc28b	Cib1
Rps9	Arpc5l	Sh3bgrl3	Spr	Uap1l1	Rpl31	Mbd3	Unc13a
Rps19	Elob	Ctsz	Comtd1	Sec61g	Camk1	Manbal	Mpv17l2
Atox1	Crip1	Drap1	Mrpl28	Eef1d	Nat9	Ssr4	Snrpd1
Wdr38	Dynll1	Lamtor2	Haus8	Blvrb	Mrpl17	Ccdc152	Krtcap2
Bst2	Wfdc17	Ndufa13	Cd300c2	Arpp19	Bola2	H2-DMb1	Bcl2a1b
Ptms	Atp6v1f	Aif1	Sqstm1	Rpl37	Mrps16	Rrp7a	Dynlrb1
S100a6	Rpl29	Use1	Smarcd3	Aurkaip1	Mir142	Rbx1	Tpt1
Rps24	Rps20	Rpl8	Fau	Selenom	Ftl1-ps1	Mydgf	Tm2d3
Ap2s1	Rps28	Psmb5	Clec2d	Gmfg	Lgals3	Ptpa	Ndufs6
Lpl	Ninj1	Lgals1	H2aj	Ndufa6	Cenpb	Bysl	Rnf187
Rplp1	Rpl35a	Ndufb9	Rbfa	Ufc1	Rps25	Wdr74	Rps10-ps2
Rps3	Cd74	Hint1	Cyb5a	Scnm1	Mrps18a	Tnfaip8l2	Cotl1
Cox7a2l	Tmem256	Arf5	Ap1s1	Nfkbia	S100a10	Nudt3	Neat1
Art3	Psmd4	Rpl38	Atp6v0e	Snx20	Tmem120a	Hcfc1r1	Pgls
Rpl35	Ccl5	Limd2	Rpl10	Rplp2	Rex1bd	Cand2	Dcaf8
Rps11	Fkbp8	Smdt1	Rps2	Rpl41	Tbcb	Tmem242	Prelid1
Ubxn11	Npc2	Cuedc2	Atf4	Aamp	Ptpn18	Ubxn1	Ndufb11
Rps15	Arrb2	Tmsb4x	Atpif1	Elof1	Aup1	Gm12411	Dnajc15
Rpl39	Rps18	Atp6v1g1	Trem2	Nhp2	Pf4	Cox5b	
Rpl18a	Ccdc12	Mettl23	Tmem160	Sod1	Tle5	Uqcrc1	
Rpl32	Eif6	Psmb1	Gadd45gip1	Ndufs7	Rpl11	Rab3il1	
Rpl14	Rps5	Tmsb10	Ms4a7	Trappc2l	Mrps26	Tkt	
Psmb6	Rpl7	Pstpip1	Rps17	Ppp4c	Trib3	Alad	
Rps8	Ubb	Ndufb1-ps	Rbfaos	Gm8292	Mien1	Gm17018	
Rplp0	D8Ertd738e	Atp5o	Mt2	Rpl37a	AI413582	Mrpl27	
Rpl13	Rps23	Rack1	Tmem192	Ier3	Yrdc	Capg	
Cstb	Cox6c	Alox5ap	Ccl4	Atp5l	Zfas1	Dok1	
Mrpl52	Cdc34	Bcat2	Tubb5	Tsr3	Naca	Tcta	
Tubb2a	Rps16	Oaz1	Gm16286	Syngr1	Slc35b2	BC031181	
Rps13	Rpl27a	Anp32a	Pde6h	Ube2m	Psmb9	Znrd1	
Rps12	Ftl1	Mt1	Bad	Selenow	Tomm7	Clec12a	
Reep5	Orai1	Hyi	Rfc2	Prorsd1		Znhit2	
Timm13	Rps3a1	Larp4b	Rpl28	Cxcl16		Fam104a	

Differentially expressed genes (DEGs) identified by DESeq2 analysis comparing glycerol-treated macrophages with vehicle control cells. Genes with Benjamini–Hochberg adjusted p-value (padj) < 0.05 were considered significantly differentially expressed, related to Fig. 3A.

Appendix Table S3. List of downregulated genes identified by RNA-seq

Downregulated gene							
Nos2	Lipa	Coro2a	Csf2rb	Sp100	Ifi47	Rnf149	Ywhag
Ifit2	Clic4	Csf2rb2	Lacc1	Stx2	Psat1	Papola	Wbp2
Cd274	Dtx3l	Cap1	Trim30a	Zfand2a	Ttyh3	Plk2	Pfkp
Thbs1	Pfkfb3	Mir6381	Tor1aip2	Baz2a	Ifi211	Pip5k1c	Kmt5a
Cd180	Itgb2	Lasp1	Cic	Ifi203	Cyren	Myo5a	Slfn5os
Slc6a6	Plxna1	Susd6	Flcn	Eef1a1	Gpnmb	Irf2bp2	Ptpn6
Tgm2	Vim	Oas3	Alcam	Gns	Bhlhe40	Zbtb7a	Phf11b
Lgals3bp	Samhd1	Golga4	Syk	Bach1	Myadm	Slc23a2	Atf3
Cmpk2	Csf2ra	B4galt5	Nono	Kansl3	Elf4	Tor3a	Fam129b
C3	Slco3a1	Med13	Mink1	Ago2	Fblim1	Ubap2l	Gvin1
Mx1	Serpinb9	Hk1	Flna	Map4	Tapbpl	Naip2	Inpp5b
Rnf213	Slc7a8	Med15	Prex1	Stat3	Sppl2a	Npc1	Lyst
Gbp5	Adap2	Nampt	Apobec1	Diaph1	Rab7b	Mknk2	Cdkn1a
Adgre1	Tns3	Nlrc5	Tpp1	Ccnd1	Tpr	Ddx5	Col1a1
Gbp4	Plec	Pacs2	Sdcbp	Etnk1	Sh3pxd2b	Mtmr6	Smg1
Gbp2	Ifi207	Mthfr	Lamp2	Spop	Eaf1	Esyt1	Phf3
Clic5	Malat1	Ctsb	Ehd4	Ankrd17	Arcn1	Zfp292	Osbpl8
Tgtp2	Ggnbp1	Basp1	Ubr4	C3ar1	Ptgs2	Glg1	Peak1
Nrp2	Myof	Fcgr1	Phf11d	Tyk2	Fndc3a	Lrp10	Gak
Parp14	Il15ra	Cpt1a	Hspa5	Ap3d1	Epb41	Ifi208	Mindy2
Gm8995	Slfn4	Rrbp1	Pabpc1	Abr	Kif1c	Ahctf1	Usp18
Irgm2	Ifi209	Ptprj	H2-Q1	Gbp6	Pla2g7	Pik3r5	Slc15a3
Sema4d	Arhgap30	Cflar	Tax1bp1	Igtp	Huwe1	Ltbp2	Igsf6
Gbp3	Lilrb4a	Mmp12	Tmem140	Tnfrsf1b	Slc9a1	Hsp90ab1	Gm5577
Ctsc	Xdh	Trim30c	Slc11a1	Rab5a	Pdcd6ip	Ifih1	Eloa
Ifi206	Oas2	Aff1	Fcgr3	Mob1a	Rasa4	Trim14	Pkp4
Oasl2	Gm4951	Herc6	Adar	Parp12	Rprd1b	Dot1l	Parp9
Pik3ap1	Csf1r	Ccdc167	Cxcl9	Nbr1	Ahnak	Ano6	Vcan
Mpeg1	Capn2	Ifit1bl1	Gna13	Hp1bp3	Hip1	Ifi204	Piezo1
Fam57b	Plin2	Cd36	Lyn	Hyou1	Ptprc	Stat6	Pdxk
Iigp1	Copa	Mfap3	Ap3b1	Cd164	Casp1	Hmga1b	Adra1a
Tln1	Hk3	Slc28a2	Iqgap1	Prdm2	Asah1	Anpep	Nup153
Il1rn	Samd9l	Jak1	Cyfip2	Casp4	Rragc	Kmt2c	Casp7
Fnip2	Slamf7	Gm5431	Kmt2d	Uba1	Creb5	Erbin	Plek
App	Tmem132a	Pml	Mertk	Emp1	Tlr8	Tbc1d9	Pcnx
Rsad2	Helz2	Siglec1	Myo1f	Oasl1	Txnip	Gnas	Man2b1
Adam8	Scpep1	Gm7336	Parp3	Dgkz	Nr3c1	Plcg2	Nckap1l
Cybb	Lrp1	Atxn2l	Igf2r	Zc3hav1	Syne1	Sec24b	Chd2
Sdc3	Ifi44	Gbp9	Slc7a2	Mgrn1	Iffo2	Csf3r	Cdk13
Mndal	Icam1	Ccrl2	Cmip	Scpep1os	Ankle2	Il1a	Ncoa4
Pgam1	Med12	Dock2	Ifi213	Zfp36l2	M6pr	Slc31a2	Calr
Znfx1	Gm15441	Adam9	Rapgef1	Usp8	Litaf	Acap2	Nfe2l2
Fbrs	Tut4	H2-T24	Spen	Tmed8	Cyfip1	Eng	Axl
Igf2bp2	Myo1e	Lrrc59	Tmem184b	Vwf	Cpeb4	Tm9sf4	Mxd1
Taok3	Fgl2	Plau	Itgb1	Klf4	Ralgds	G6pdx	Il7r
Stat2	Map2k1	Hmga1	Arel1	Prkx	Pxk	Cd86	Leng8
Msn	Irgm1	Tlr9	Lgals8	Vegfa	Nsd3	Fam20b	Ankrd52
Zmiz1	Rgl1	Lgals9	Tmem104	Dennd4c	Smurf1	Serinc3	Hsp90aa1
Myh9	Fyb	Polr2a	Slamf8	Ankrd11	Rnf10	Numb	Zswim4
	Wasf2		Tmcc3		Hpgds		Parp11

Differentially expressed genes (DEGs) identified by DESeq2 analysis comparing glycerol-treated macrophages with vehicle control cells. Genes with Benjamini–Hochberg adjusted p-value (padj) < 0.05 were considered significantly differentially expressed, related to Fig. 3A.

Appendix Table S4. Gene list of mitochondrial metabolic pathways for heatmap visualization shown in Figure 3B

Gene	p-val	ETC Complex affiliation
Ndufa10	0.0047	Complex I
Ndufv1	0.0554	Complex I
Uqcc3	0.0134	Complex III
Ndufb11	0.0240	Complex I
Ndufa8	0.0557	Complex I
Uqcrh	0.0948	Complex III
Ndufv2	0.0570	Complex I
Ndufa4	0.0303	Complex I
Uqcrc1	0.0222	Complex III
Ndufa13	0.0287	Complex I
Uqcr10	0.0147	Complex III
Ndufb3	0.0133	Complex I
Ndufc1	0.0710	Complex I
Ndufa9	0.0785	Complex I
Ndufa2	0.0004	Complex I
Ndufb1-ps	0.0014	Complex I
Ndufb9	0.0103	Complex I
Ndufa6	0.0364	Complex I
Ndufb6	0.0074	Complex I
Uqcr11	0.0039	Complex III
Uqcrq	0.0033	Complex III
Ndufs7	0.0130	Complex I
Ndufa7	0.0150	Complex I
Ndufb4	0.3720	Complex I
Ndufb8	0.3397	Complex I
Uqcrfs1	0.9205	Complex III
Ndufs3	0.3186	Complex I
Ndufv3	0.1933	Complex I
Ndufs4	0.3068	Complex I
Uqcrb	0.0945	Complex III
Sdhb	0.0377	Complex II
Ndufb7	0.0733	Complex I
Ndufs6	0.0411	Complex I
Ndufa3	0.0459	Complex I
Ndufa1	0.0473	Complex I
mt-Co1	0.4217	Complex IV
mt-Nd5	0.9949	Complex I
mt-Nd2	0.7073	Complex I
mt-Nd4	0.5369	Complex I
mt-Nd1	0.1879	Complex I
Uqcrc2	0.1031	Complex III
Sdhd	0.0937	Complex II

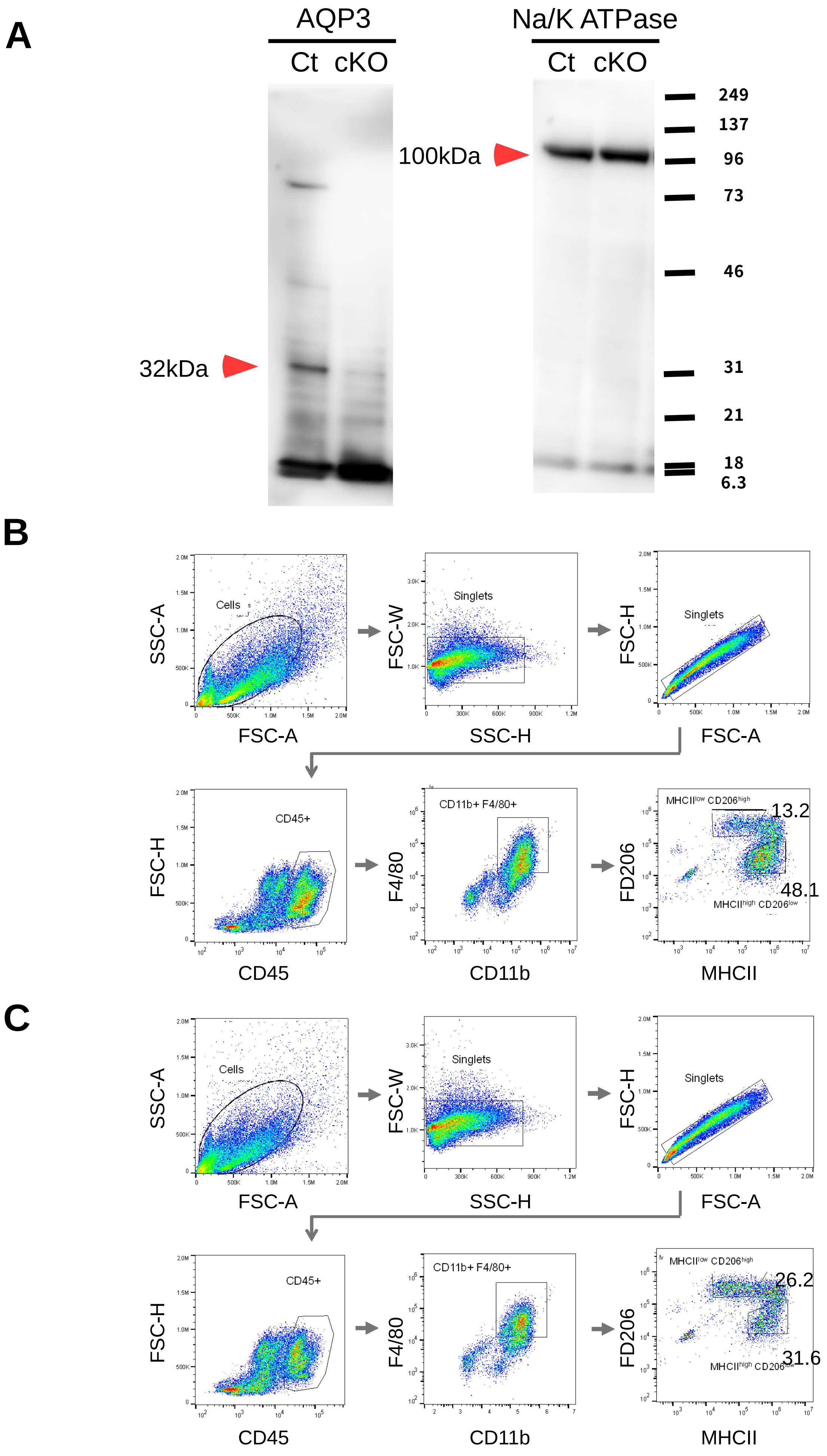

Appendix Figure S1.

(**A**) Western blotting as uncropped images (related to Figure 4D). Immunoblotting of the membrane-rich fraction from control and AQP3 cKO BMDMs with anti-AQP3 and anti-Na +/K+-ATPase antibodies.

(**B, C**) Example flow cytometry gating strategy for quantifying macrophage populations from digested adipose tissues. (**B**) Control mice with HFD. (**C**) AQP3 cKO mice with HFD.
