# Supplementary material for: Glycerol enhances mitochondrial metabolism and inflammatory response in pro-inflammatory macrophages
Source: EMBO Rep. 2026 Apr 14;27(10):2614–38. doi: 10.1038/s44319-026-00747-y (PMC13219733; doi:10.1038/s44319-026-00747-y)
Supplement: Supplementary file 13 — Expanded View Figures [file 44319_2026_747_MOESM13_ESM.pdf]

Expanded View Figures

Figure EV1. Effect of FBS on macrophage polarization.

(**A**) Bone marrow cells from C57BL/6 mice were incubated with M-CSF (10 ng/ml) for seven days (M0 macrophages), then treated with LPS (10 ng/ml, 24 h) for M1-type polarization. The indicated mRNA expression levels were quantified by qPCR. Data were expressed as the ratio of 18 s RNA ($n = 4$–5). (**B**) Glycerol contents in FBS, dialyzed FBS (dFBS), and serum-free RPMI medium were measured using the high-sensitivity free glycerol fluorometric assay kit (Sigma-Aldrich, Cat# MAK270). (**C**) LPS-primed BMDMs were incubated in RPMI medium supplemented with FBS, dFBS, or no FBS for 24 h. The indicated mRNA expression levels were quantified by qPCR ($n = 6$). (**D**) M0-type BMDMs were stimulated with LPS (10 ng/mL) in medium containing FBS, dFBS, or serum-free medium for 6 h. The indicated mRNA expression levels were quantified by qPCR ($n = 6$). Data information: All data presented in Fig. EV1 are mean ± standard error of the mean (SEM). *N* values indicate biological replicates. (**A**) Unpaired *t*-tests. (**C**) One-way ANOVA with Sidak's multiple comparison test. (**D**) One-way ANOVA with Dunnett's multiple comparison test. Exact P values are reported, except where the adjusted P value was smaller than 0.0001, in which case it is reported as $P < 0.0001$. Source data are available online for this figure.

 © The Author(s)

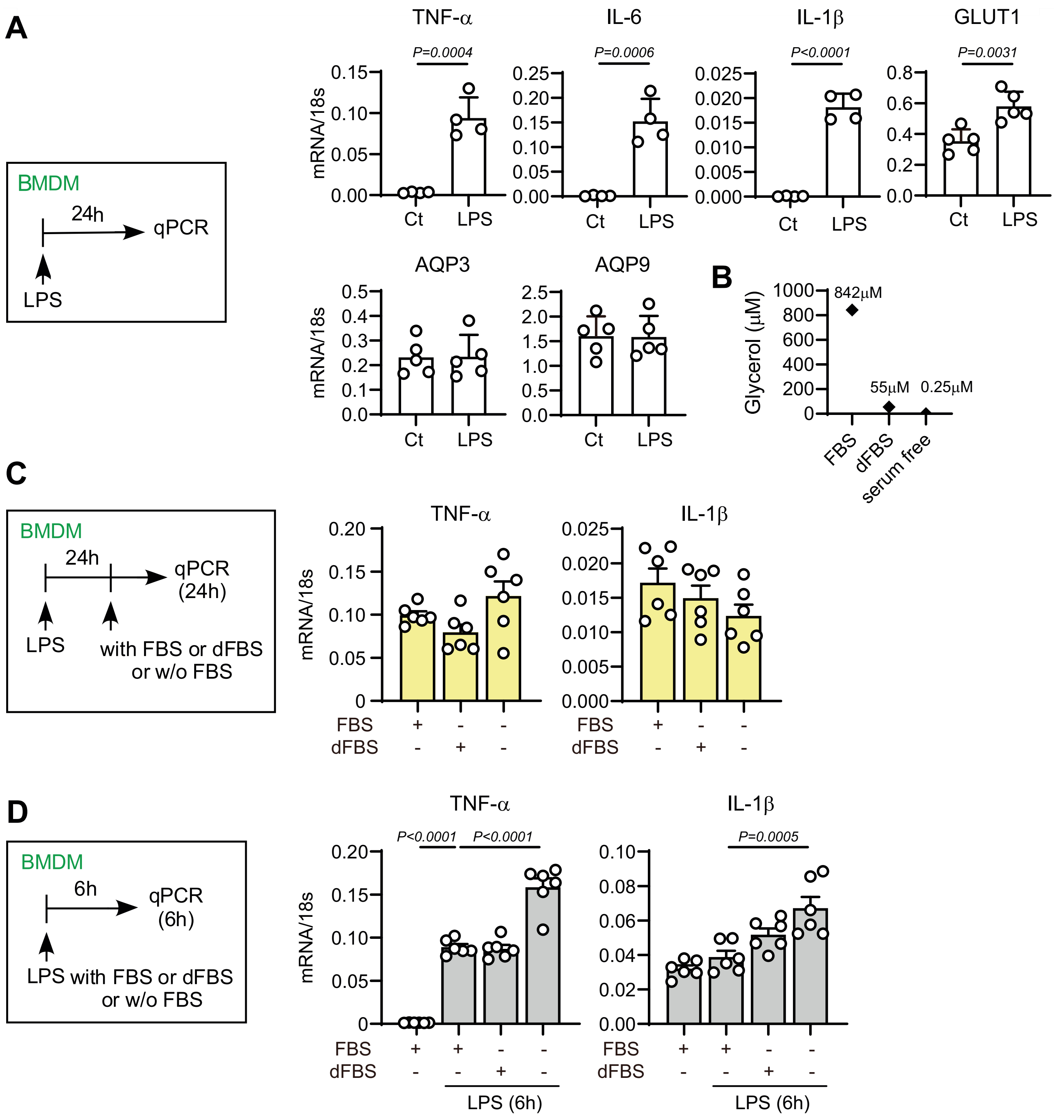
A
BMDM
24h
qPCR
LPS
TNF-α
P=0.0004
IL-6
P=0.0006
IL-1β
P<0.0001
GLUT1
P=0.0031
mRNA/18s
Ct
LPS
AQP3
AQP9
B
Glycerol (μM)
842μM
55μM
0.25μM
FBS
dFBS
serum free
C
BMDM
24h
qPCR
(24h)
LPS
with FBS or dFBS
or w/o FBS
TNF-α
IL-1β
FBS
dFBS
D
BMDM
6h
qPCR
(6h)
LPS with FBS or dFBS
or w/o FBS
TNF-α
P<0.0001
P<0.0001
IL-1β
P=0.0005
LPS (6h)

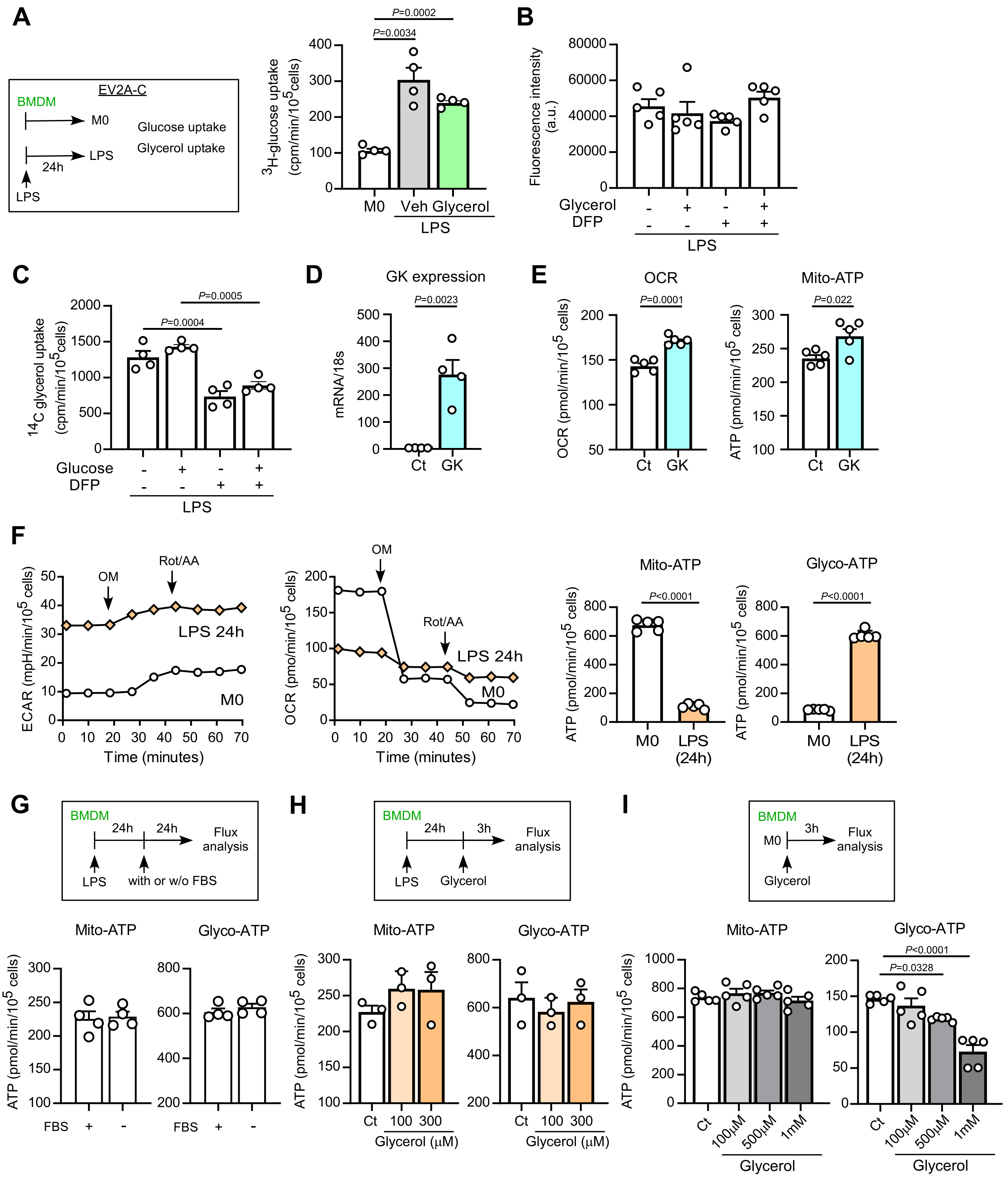
A
EV2A-C
BMDM
M0
LPS
24h
LPS
Glucose uptake
Glycerol uptake
P=0.0002
P=0.0034
3H-glucose uptake (cpm/min/10^5cells)
M0
Veh
Glycerol
LPS
B
Fluorescence intensity (a.u.)
Glycerol
DFP
LPS
C
P=0.0005
P=0.0004
14C glycerol uptake (cpm/min/10^5cells)
Glucose
DFP
LPS
D
GK expression
P=0.0023
mRNA/18s
Ct
GK
E
OCR
P=0.0001
OCR (pmol/min/10^5 cells)
Mito-ATP
P=0.022
ATP (pmol/min/10^5 cells)
Ct
GK
F
OM
Rot/AA
LPS 24h
M0
ECAR (mpH/min/10^5 cells)
OCR (pmol/min/10^5 cells)
Time (minutes)
Mito-ATP
P<0.0001
Glyco-ATP
P<0.0001
ATP (pmol/min/10^5 cells)
M0
LPS (24h)
G
BMDM
24h
24h
Flux analysis
LPS
with or w/o FBS
Mito-ATP
Glyco-ATP
ATP (pmol/min/10^5 cells)
FBS
H
BMDM
24h
3h
Flux analysis
LPS
Glycerol
Mito-ATP
Glyco-ATP
Ct
100
300
Glycerol (μM)
I
BMDM
M0
3h
Flux analysis
Glycerol
Mito-ATP
Glyco-ATP
P<0.0001
P=0.0328
Ct
100μM
500μM
1mM
Glycerol

 © The Author(s)

Figure EV2. Glycerol does not affect glucose uptake.

(**A**) [^{3}H]-glucose uptake for one hour in M0 and LPS-primed macrophages in the presence or absence of glycerol (1 mM). Cells were starved of glucose for at least one hour prior to the assay ($n = 4$). (**B**) LPS-primed macrophages were starved of glucose for at least one hour prior to the assay. Glucose uptake for 1 h was measured using a Glucose Uptake Assay Kit (DOJINDO, UP02) with glycerol (1 mM) and/or DFP00173 (1 mM) ($n = 5$). (**C**) [^{14}C]-glycerol uptake in LPS-primed macrophages. To assess whether glycerol uptake depends on intracellular glucose levels, cells were maintained in glucose-depleted medium for three hours prior to the assay, followed by incubation with [^{14}C]-glycerol for 3 min ($n = 4$). (**D**, **E**) BMDMs were overexpressing GK by adenovirus infection. (**D**) mRNA encoding for GK. Data were expressed as the ratio to 18 s RNA ($n = 4$). (**E**) Cellular OCR and mito-ATP production by ATP Rate Assay Kit in GK overexpressed LPS-primed macrophages in the presence of glycerol (1 mM) ($n = 5$). (**F**) BMDMs (M0) and LPS-primed BMDMs (M1) from wild-type mice were assessed using an ATP Rate Assay Kit with a Flux analyzer. (left) ECAR and OCR. (right) Mitochondria-derived and glycolysis-derived ATP production ($n = 5$). (**G**) LPS-primed BMDMs were incubated in medium supplemented with FBS or without FBS for 24 h. Mitochondria-derived and glycolysis-derived ATP production were analyzed using an ATP Rate Assay Kit with a Flux analyzer ($n = 4$). (**H**) LPS-primed BMDMs were incubated with glycerol (100 or 300 μM) in serum-free medium for 3 h. Mitochondria-derived and glycolysis-derived ATP production were analyzed using an ATP Rate Assay Kit with a Flux analyzer ($n = 3$). (**I**) M0-type BMDMs were incubated with glycerol (100, 500 μM or 1 mM) in serum-free medium for 3 h. Mitochondria-derived and glycolysis-derived ATP production were analyzed using an ATP Rate Assay Kit with a Flux analyzer ($n = 5$). Data information: All data presented in Fig. EV2 are mean ± standard error of the mean (SEM). *N* values indicate biological replicates. (**A–C**) One-way ANOVA with Sidak's multiple comparison test. (**D–G**) Unpaired *t*-tests. (**H**, **I**) One-way ANOVA with Dunnett's multiple comparison test. Exact *P* values are reported, except where the adjusted *P* value was smaller than 0.0001, in which case it is reported as $P < 0.0001$. Source data are available online for this figure.

© The Author(s)

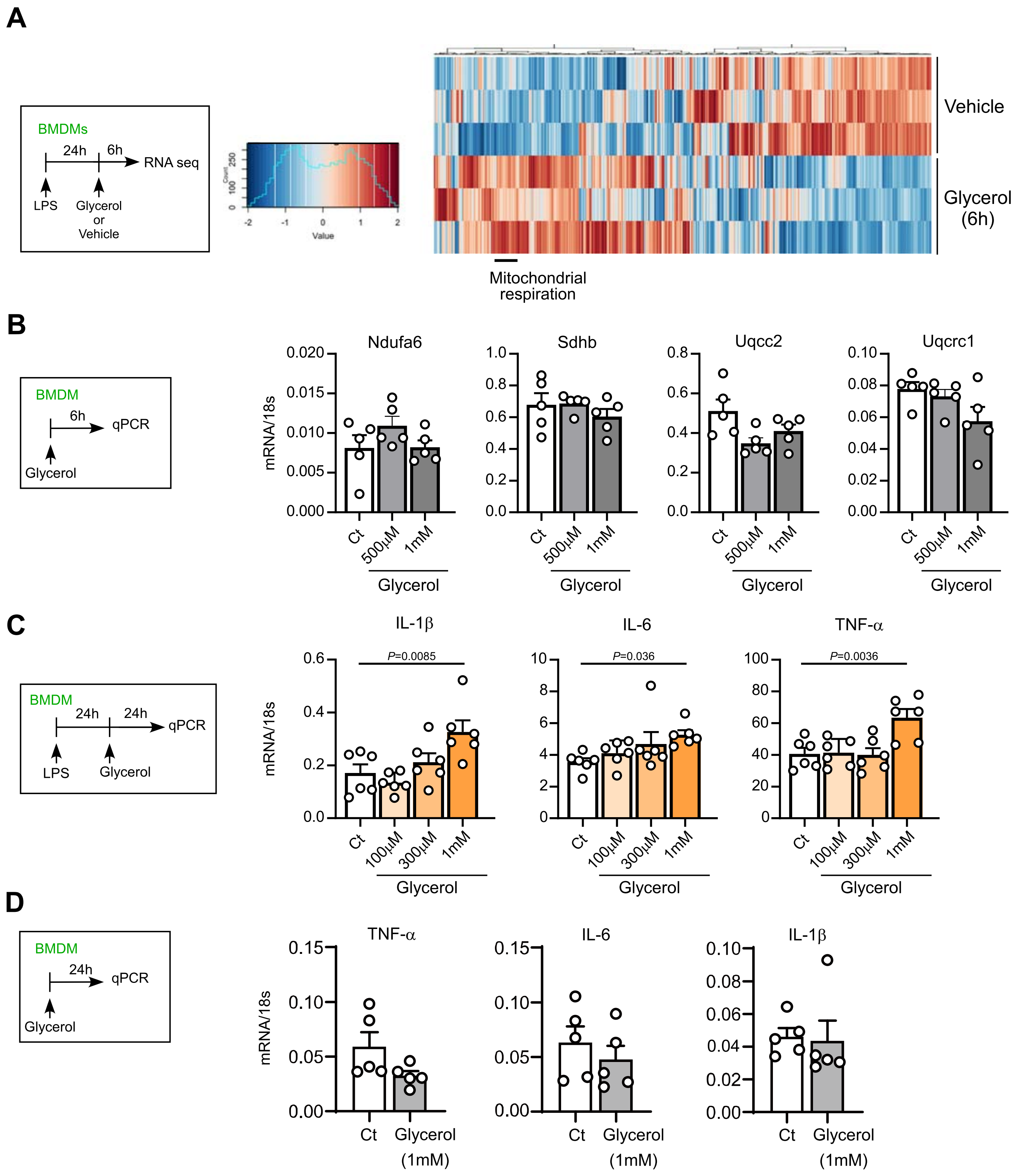
A
BMDMs
24h
6h
RNA seq
LPS
Glycerol
or
Vehicle
Value
Vehicle
Glycerol
(6h)
Mitochondrial
respiration
B
BMDM
6h
qPCR
Glycerol
Ndufa6
Sdhb
Uqcc2
Uqcrc1
mRNA/18s
Ct
500μM
1mM
Glycerol
C
BMDM
24h
24h
qPCR
LPS
Glycerol
IL-1β
IL-6
TNF-α
P=0.0085
P=0.036
P=0.0036
mRNA/18s
Ct
100μM
300μM
1mM
Glycerol
D
BMDM
24h
qPCR
Glycerol
TNF-α
IL-6
IL-1β
mRNA/18s
Ct
Glycerol
(1mM)

 © The Author(s)

Figure EV3. Glycerol alters gene expression in pro-inflammatory macrophages.

(**A**) LPS-primed BMDMs were stimulated with glycerol (1 mM) for 6 h after glycerol starvation. RNA sequencing analysis was conducted, as shown in Fig. 3A. Differentially expressed genes (Log_2 [fold change] ≤−2 or ≥2) in control and glycerol-treated cells as summarized by heatmap. Red = upregulated; blue = downregulated ($n = 3$, biologically independent samples). (**B**) M0-type BMDMs were incubated with glycerol (500 μM or 1 mM) in serum-free medium for 6 h. The indicated mRNA expression levels were quantified by qPCR ($n = 5$). (**C**) LPS-primed BMDMs were incubated with glycerol (100, 300 μM or 1 mM) in serum free medium for 24 h. The indicated mRNA expression levels were quantified by qPCR ($n = 6$). (**D**) M0-type BMDMs were incubated with glycerol (1 mM) in serum free medium for 24 h. The indicated mRNA expression levels were quantified by qPCR ($n = 5$). Data information: All data presented in Fig. EV3 are mean ± standard error of the mean (SEM). *N* values indicate biological replicates. (**B**, **C**) One-way ANOVA with Dunnett's multiple comparison test. (**D**) Unpaired *t*-tests. Exact *P* values are reported. Source data are available online for this figure.

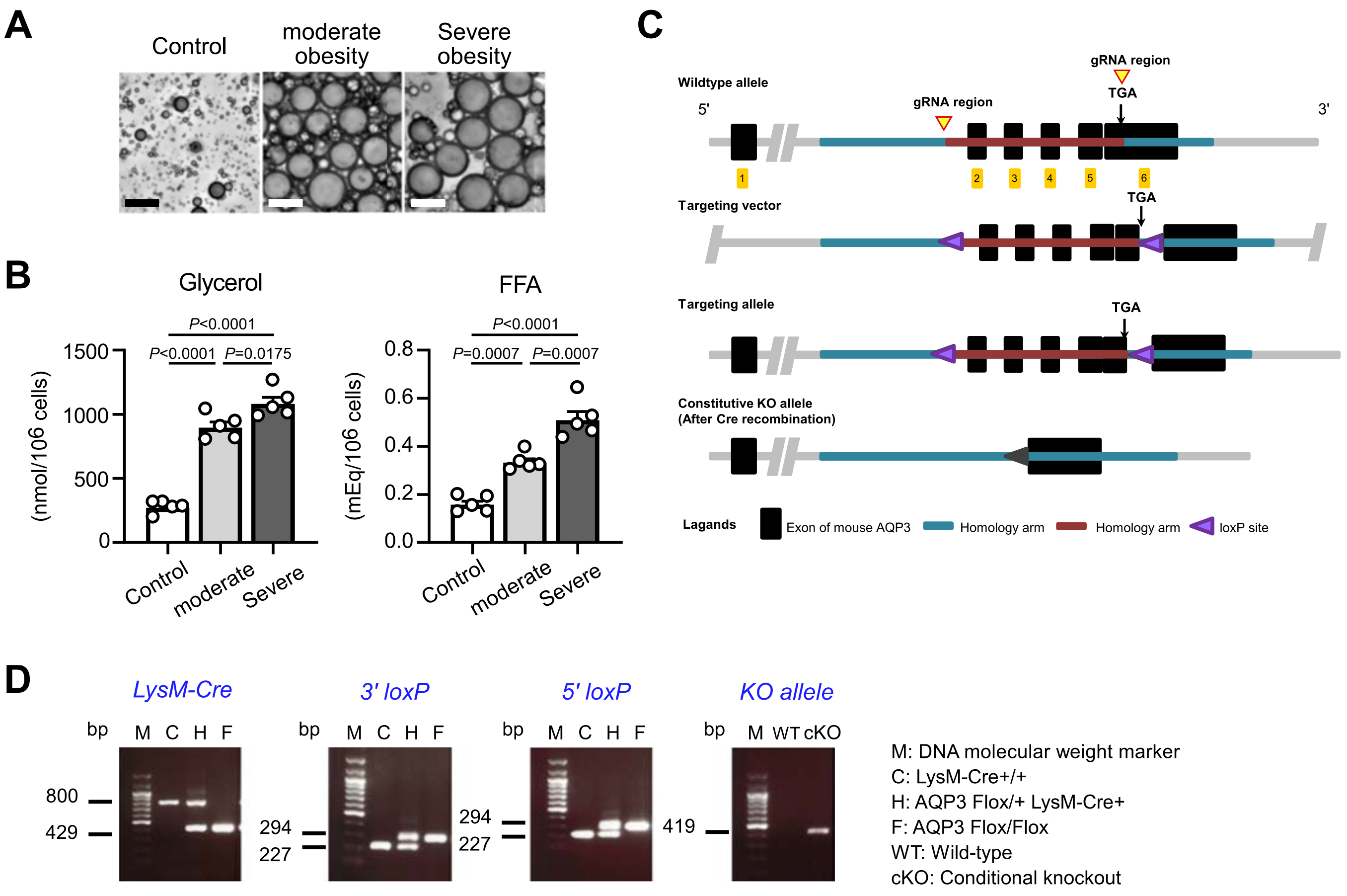

Figure EV4. Generation of macrophage-specific AQP3 conditional knockout mice.

(**A**) Images of adipocytes from control (NC) and HFD-induced obese mice (moderate: ~35-g weight, severe: ~50-g weight). (**B**) Glycerol and non-esterified fatty acid (FFA) content in the culture medium ($n = 5$). (**C**) Generation of AQP3$^{flox/flox}$ mice. Overview of the targeting strategy. (**D**) Representative detection of AQP3 deletion in AQP3$^{flox/flox}$ LysM-Cre^{+} (AQP3 cKO) by PCR with genomic DNA. Data information: All data presented in Fig. EV4 are mean ± standard error of the mean (SEM). *N* values indicate biological replicates. (**B**) One-way ANOVA with Tukey's multiple comparisons test. Exact *P* values are reported, except where the adjusted *P* value was smaller than 0.0001, in which case it is reported as $P < 0.0001$. Source data are available online for this figure.

 © The Author(s)

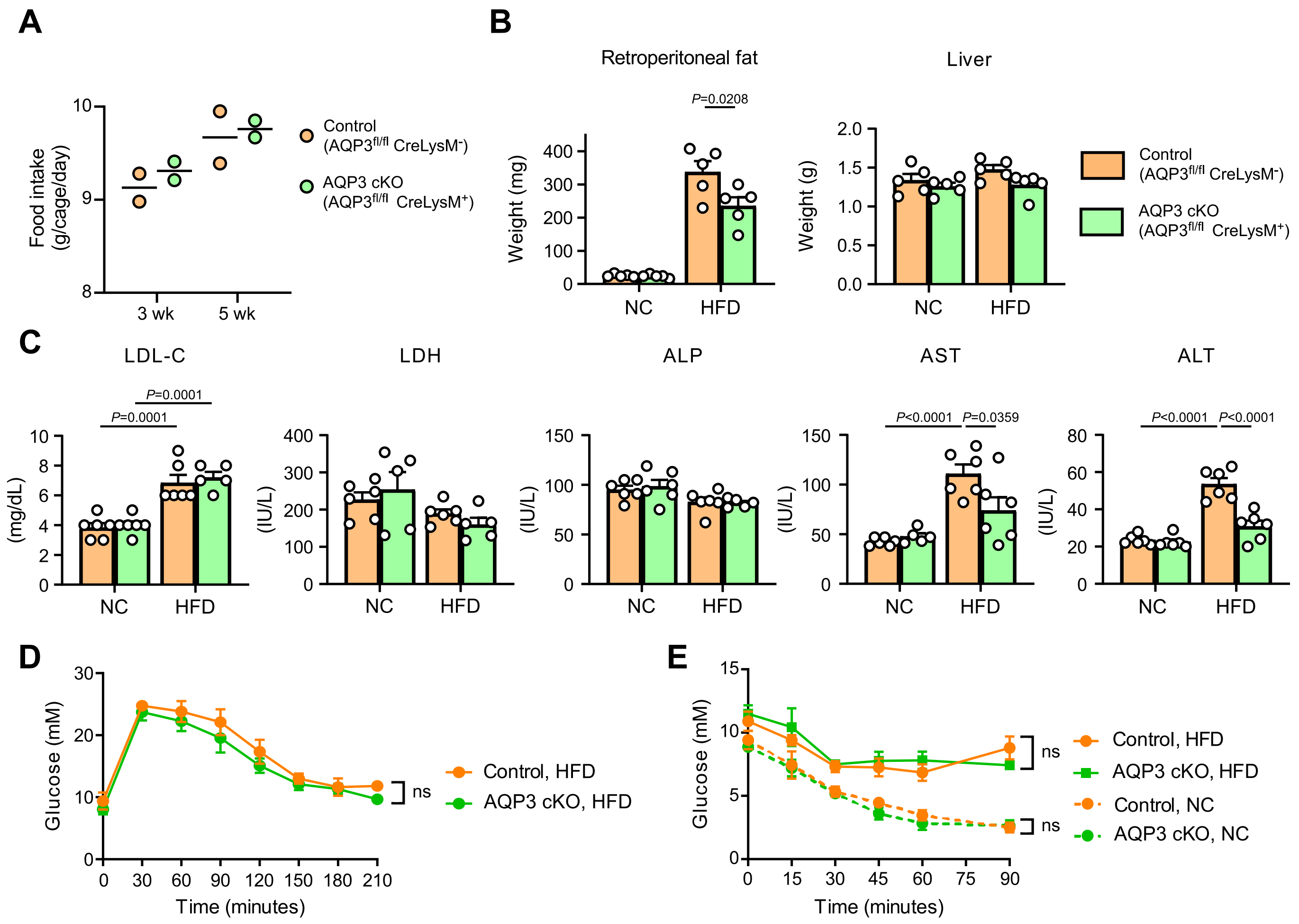

Figure EV5. AQP3 deficiency in macrophages attenuates HFD-induced obesity.

(**A–E**) AQP3 cKO and control mice were fed high fat diet (HFD) or normal chow (NC) for 6 weeks, same setting as shown in Fig. 5. (**A**) HFD chow consumption per cage for 24 h was measured at 3 and 5 weeks, demonstrating no difference between the two groups (two cages for each condition, three mice in each cage). (**B**) Weight of adipocyte tissues and liver ($n = 5$). (**C**) Low-density lipoprotein cholesterol (LDL-C), lactate dehydrogenase (LDH), alkaline phosphatase (ALP), aspartate aminotransferase (AST), and alanine aminotransferase (ALT) contents in serum ($n = 5$–6). (**D**) Glucose tolerance test in control and AQP3 cKO mice fed with HFD ($n = 3$). (ns not significant). (**E**) Insulin tolerance test in control and AQP3 cKO mice fed with NC or HFD ($n = 5$). (ns not significant). Data information: All data presented in Fig. EV5 are mean ± standard error of the mean (SEM). *N* values indicate biological replicates. (**B–D**) Two-way ANOVA with Sidak's multiple comparisons test. (**E**) Two-way ANOVA with Tukey's multiple comparisons test. Exact *P* values are reported, except where the adjusted *P* value was smaller than 0.0001, in which case it is reported as $P < 0.0001$. Source data are available online for this figure.

© The Author(s)
